# Supplementary material for: A fast numerical method for oxygen supply in tissue with complex blood vessel network
Source: PLoS One. 2021 Feb 26;16(2):e0247641. doi: 10.1371/journal.pone.0247641 (PMC7909958; doi:10.1371/journal.pone.0247641)
Supplement: Attachment — Submitted filename: Review_Paper_Oxygen_2020.pdf [file pone.0247641.s003.pdf]

PONE-D-20-33706

A fast numerical method for oxygen supply in tissue with complex blood vessel network

by Y. Lu, D. Hu and W. Ying

In the submitted paper, the authors present a mathematical model for oxygen supply of living tissue via a blood vessel network. The mathematical model is given by an elliptic diffusion reaction equation in three dimensions (3D) yielding the partial pressure of oxygen in tissue, while the partial pressure of oxygen within the blood vessels is represented by an ordinary differential equation (ODE or 1D PDE) called Hill's equation.

In order to couple both equations suitable source terms for both equations are established, where the source term for the 3D PDE is given by a line source term. The specific shape of the line source term is governed by the midlines of the blood vessels. Solving such PDE systems numerially is a challenging task, since the solution in 3D exhibits singularities along the line. As a consequence the mesh around the lines has to be sufficiently fine, which increases the numerical effort in particular in 3D.

To circumvent this drawback, the authors propose a post processing to correct the numerical errors occuring in context of a coarse mesh. The arsing linear systems of equations are solved in an iterative way. The performance and precision of this method are illustrated by means of several tests. Essentially, the paper is well written and organized. However, there are some points that have to be improved before the paper can be published. All in all, I would recommend a **minor revision** for the submitted paper. My specific remarks are listed below.

Remarks:

1. The authors motivate the objective of their work by means of angiogenesis. However, there is another application area for models that can be used to simulate oxygen supply of tissue: Generation of artifical microvascular networks. To obtain further information on this topic one could consider e.g. the following publications:

   – T. Köppl, E. Vidotto & B. Wohlmuth (2020). A 3D-1D coupled blood flow and oxygen transport model to generate microvascular networks. International Journal for Numerical Methods in Biomedical Engineering, DOI: 10.1002/cnm.3386

   – M. Schneider, J. Reichold, B. Weber, G. Szekely & S. Hirsch (2012). Tissue metabolism driven arterial tree generation. Medical image analysis, 16(7), 1397-1414.

2. In Section 2.2., blood flow and blood pressure are modeled by means of the Poiseuille model without taking the exchange with the surrounding tissue into account using e.g. Starling's law. Please justify, why the fluid exchange through the porous vessel wall is omitted.

3. In Section 2.4, the authors model the oxygen exchange between the tissue and the vascular system by means of the averaged PO_2 gradient in 3D. However in several publications e.g.:

   L. Cattaneo & P. Zunino (2014). A computational model of drug delivery through microcirculation to compare different tumor treatments. International journal for numerical methods in biomedical engineering, 30(11), 1347-1371.

   the exchange of substances like oxygen between the vascular system and tissue is modeled by the Kedem-Katalchsky law, which is a standard filtration law for permeable membranes. Why did the authors consider the exchange terms in Section 2.4 and not the Kedem-Katalchsky's law?

4. Section 3.2: Why do the authors use Finite Differences to discretize the diffusion reaction equation? It is a well-known fact that Finite Differences are locally not mass conservative, which is an important feature of a numerical method applied to a flow or transport problem.

4. In Section 3.3 the authors suggest a post-processing method to reduce the errors caused by a coarse mesh. In this context, I would like to ask the authors to mention and briefly discuss a recent publication on the numerical modeling of 3D-1D coupled blood flow problems:

   T. Koch, M. Schneider, R. Helmig & P. Jenny (2020). Modeling tissue perfusion in terms of 1d-3d embedded mixed-dimension coupled problems with distributed sources. Journal of Computational Physics, 410, 109370.

5. Section 5.2: How do the authors compute the error between two levels? What norms are used? Furthermore I would suggest to report a suitable norm of the solution for several refinement levels. By this, the reader can observe on which mesh the numerical solution is representative.

6. Section 5.4, Figure 5: From my point of view the assignment of the labels a)-d) in the caption of Figure 5 are wrong. In a) you show the full network geometry, while in b)-d) the oxygen profiles for different retina depths are shown.

7. Section 6: How do the authors know that if the numerical error is below 1 %, it is smaller than the modeling error? How can the modeling error

be quantified? Please provide a reference for this claim.

8. Appendix A: Lines 448/449: What are the boundary conditions and source terms for the Laplace equation? Do you consider the fundamental solution of the Laplace equation for the Taylor expansion?

9. Appendix B: Is the provided ϕ really continuous? Performing some computations reveals that the provided ϕ is not continuous at $r = 1$ and $r = -1$. Is the formula for ϕ correct?

10. There are several typos and language errors. Thus I would recommend a proof reading to improve the quality of the written English.
