# Supplementary material for: A fast numerical method for oxygen supply in tissue with complex blood vessel network
Source: PLoS One. 2021 Feb 26;16(2):e0247641. doi: 10.1371/journal.pone.0247641 (PMC7909958; doi:10.1371/journal.pone.0247641)
Supplement: Attachment — Submitted filename: Responses to reviewer.docx [file pone.0247641.s002.docx]

We appreciate the comments of the reviewer which are important for us to improve our work and the manuscript. We have made significant improvements following the suggestions of the reviewer. Detailed responses to the reviewer are listed in the following. The reviewer’s comments are quoted in Italic (blue).

Reviewer’s comment:

*The authors consider the standard model for the oxygenation of living tissue through a blood vessel network and present a numerical method to compute the oxygen concentration field. They apply their algorithm to a two-dimensional vessel network of a mouse retina and analyze its numerical efficiency.*

Our numerical method can be naturally extended to three-dimensional simulations. In the revised manuscript, we have applied three-dimensional simulations in a domain with a three-layers retina vessel networks. Corresponding results are shown in Section 5, highlighted in green.

*Reviewer’s comment:*

*Since the model for oxygenation through a blood vessel network as well as the simplifications described in section 2.5 have been discussed and analyzed extensively in the literature (ref. 24, 17, 25) the only new aspect of the present work appears to be the iteration scheme discussed in section 3 for solving the nonlinear equations of the model. The authors claim that this is fast and accurate, but no comparison with experimental data or with other existing algorithms is made.*

We have added a subsection (Section 5.4 and Figure 5, 6, and 7, highlighted in green) to show the comparison results of our three-dimensional simulation results on mice retina with previous experimental and simulation results. Despite the large differences in the independent experimental results, which may be induced by different parameter settings and experimental conditions, the oxygen profiles indeed share similar features, e.g., relatively high oxygen partial pressure is observed near the choroid surface and the deep capillary plexi.

In this section, we have also added a section to show the consistency between our numerical results and that obtained with the method developed in Ref. 17.

*Reviewer’s comment*

*The example network used by the authors contains only 4096 vessel segments and is two-dimensional, which is very small compared to state of the art implementations of oxygen supply computations, as for instance the computational study of a fully vascularized tissue with a volume of around 1cm^3 containing around 10^6 micro-vessel-segments, published in Welter etal PLOS ONE 11 e0161267 (2016). Since an efficiency gain is claimed by the authors a performance check against such earlier implementations must be performed.*

Thank the reviewer for reminding us the work of Welter et al. We have added three-dimensional simulation results for an entire mouse retina vascular system in a cube with edge length of 4.4mm, using a 512*512*512 mesh. This mesh can also be used for a 1cm^3 cube with step size h=20μm. In this case, according to our numerical results, the corresponding numerical error is about 4% (see Figure 8(a)).

We have not performed a 3-D simulation of fully vascularized tissue due to lack of suitable experiment data on 3-D vascular structure. However, in our numerical method, the time cost associated with blood vessels (the OP-time defined in the main text) increases linearly with the total length of blood vessels (Detail numerical analysis is included in Section 5.6, highlighted in green). According to our estimation, the time cost associated with blood vessels for a fully vascularized tissue is (much) smaller than that for solving the three-dimensional PDE for oxygen field when h<20μm. Therefore, our method can be used for fully vascularized tissue domain without significant increase in total time cost.

According to our numerical results, a step size of h=10μm can be used to achieve about 1% numerical error. In this case, a simulation in a 1cm^3 fully vascularized tissue domain can be achieved within 20 hours on a standard PC.

Corresponding analysis and results are included Section 5.6, highlighted in green.

*Reviewer’s comment*

*Moreover, the accuracy of the results obtained by the computation should be checked by comparing the resulting oxygen concentration field quantitatively with the predictions obtained by other methods. To make such a check the authors could use, for instance, the software package Tumorcode, publicly available on github and described in Fredrich etal, Europ. Phys. J. E 41 55 (2018). This is actually the program with which the results of Welter etal mentioned above have been obtained. Finally also a comparison of the results with experimental data of oxygen partial pressures inside and outside vessels should be made.*

As mentioned above, we have added the comparison with experimental and simulation results based on our three-dimensional simulation results.

*Reviewer’s comment:*

*Since neither the performance gain nor the accuracy of the algorithm presented by the authors is critically discussed I doubt that the main findings of the manuscript represent a real progress in comparison to existing work. Therefore I cannot recommend the publication of his manuscript in PLOS ONE.*

In the revised manuscript, we have added the numerical method and simulation results for three-dimensional simulations. A 512*512*512 mesh is used for simulation of mice retina. This mesh can also be used in simulation of a 1 cm^3 simulation with h=20μm. The time cost for the simulation in a fully vascularized 1cm^3 tissue with step size h=10μm is also estimated, in which we can expect a relative numerical error of about 1%. We would also like to point out that the nonlinear iteration numbers (thus the simulation time) can be significantly reduced if we just expect a 10% numerical error.
